# Retinoic acid modulation of granule cell activity and spatial discrimination in the adult hippocampus

**DOI:** 10.3389/fncel.2024.1379438

**Published:** 2024-04-17

**Authors:** Yun-Gwon Yeo, Jeongrak Park, Yoonsub Kim, Jong-Cheol Rah, Chang-Hoon Shin, Seo-Jin Oh, Jin-Hyeok Jang, Yaebin Lee, Jong Hyuk Yoon, Yong-Seok Oh

**Affiliations:** ^1^Department of Brain Sciences, Daegu Gyeongbuk Institute of Science and Technology (DGIST), Daegu, Republic of Korea; ^2^Sensory and Motor Systems Research Group, Korea Brain Research Institute (KBRI), Daegu, Republic of Korea; ^3^Neurodegenerative Diseases Research Group, Korea Brain Research Institute (KBRI), Daegu, Republic of Korea; ^4^Emotion, Cognition and Behavior Research Group, Korea Brain Research Institute (KBRI), Daegu, Republic of Korea

**Keywords:** retinoic acid, vitamin A, hippocampal neuroplasticity, dentate gyrus, granule cells, spatial discrimination

## Abstract

Retinoic acid (RA), derived from vitamin A (retinol), plays a crucial role in modulating neuroplasticity within the adult brain. Perturbations in RA signaling have been associated with memory impairments, underscoring the necessity to elucidate RA’s influence on neuronal activity, particularly within the hippocampus. In this study, we investigated the cell type and sub-regional distribution of RA-responsive granule cells (GCs) in the mouse hippocampus and delineated their properties. We discovered that RA-responsive GCs tend to exhibit a muted response to environmental novelty, typically remaining inactive. Interestingly, chronic dietary depletion of RA leads to an abnormal increase in GC activation evoked by a novel environment, an effect that is replicated by the localized application of an RA receptor beta (RARβ) antagonist. Furthermore, our study shows that prolonged RA deficiency impairs spatial discrimination—a cognitive function reliant on the hippocampus—with such impairments being reversible with RA replenishment. In summary, our findings significantly contribute to a better understanding of RA’s role in regulating adult hippocampal neuroplasticity and cognitive functions.

## 1 Introduction

Retinoic acid (RA), a derivative of vitamin A (VA), acts as a ligand for nuclear RA receptors (RARs) (Shearer et al., 2012), which are crucial in chordate development (Mendelsohn et al., 1994). The RAR family consists of three isoforms—RARα, RARβ, and RARγ—that pair with retinoid X receptors (RXRα, RXRβ, RXRγ) to form heterodimers. These complexes bind to RA-responsive elements (RAREs) within gene promoter regions, influencing gene transcription by recruiting nuclear receptor coactivators or corepressors (Cunningham and Duester, 2015). Chromatin immunoprecipitation assays using RAR antibodies have identified up to 15,000 potential RAREs across the mouse genome (Moutier et al., 2012).

Beyond its role in neurodevelopment, RA is increasingly recognized for its regulatory capacity in adult neuroplasticity across various brain regions (Chen et al., 2014; Lenz et al., 2021b). Research involving RARα conditional knockout models has linked RA signaling to spine maturation and synaptic regulation in the pyramidal neurons of the somatosensory cortex (Yee and Chen, 2016; Zhong et al., 2018). Furthermore, RA’s contribution to cognitive and emotional functions has been supported by vitamin A depletion studies (Hsu et al., 2019; Huang and Chen, 2020), which have also highlighted its importance in spatial learning and memory (Cocco et al., 2002; Jiang et al., 2012). Disturbances in RA signaling have been associated with a range of neuro-pathologies, including schizophrenia (Reay et al., 2020), epilepsy (Sayyah et al., 2005), depression (Hu et al., 2016; Huang and Chen, 2020), and Alzheimer’s disease (Corcoran et al., 2004). Notably, while RA infusion in the basolateral amygdala presents antiepileptic properties (Sayyah et al., 2005), chronic administration can lead to depressive-like states through hyperactivity in the hypothalamic-pituitary-adrenal axis (Huang and Chen, 2020). Nonetheless, the precise neural mechanisms that RA influences behavior remain poorly understood in health and disease.

The hippocampus, comprising distinct subregions such as CA1–3 and dentate gyrus (DG), is pivotal for memory encoding and retrieval (Knierim, 2015). The DG, in particular, is critical for encoding spatial and contextual information upon exposure to a novel environment (NE) (Lisman et al., 2005; Wiltgen et al., 2010). This sparse activation of specific DG granule cells (GCs) forms what is known as the memory engram (Nitz and McNaughton, 2004). This memory engram in the DG is substantial for cognitive pattern separation (Josselyn et al., 2015; Perusini et al., 2017), enabling the accurate differentiation between similar spatial and contextual conditions (Kee et al., 2007; Guo et al., 2018). While optogenetic activation of these engram cells can trigger memory recall, their inhibition can obstruct memory processes altogether (Liu et al., 2012; Lamothe-Molina et al., 2022). Conversely, excessive GC activity can disrupt memory precision, as observed in epileptic models where abnormally heightened GC activation impedes engram formation and pattern separation (Pekcec et al., 2008; Hester and Danzer, 2014).

Retinoic acid is primarily stored in the liver and is regulated to ensure stable levels in adults (Shearer et al., 2012). Within the brain, the hippocampus is a key region for RA synthesis and degradation (Wołoszynowska-Fraser et al., 2020). RA signaling through RARs and RXRs is crucial for modulating adult hippocampal neuroplasticity (Misner et al., 2001; Vesprini and Spencer, 2014; Park et al., 2018). Despite previous findings about RA-responsive cells in the adult hippocampus (McCaffery et al., 2006; Goodman et al., 2012), the properties of these cells and their role in memory processes have not been fully determined. Our present study profiles the cell type-specific and sub-regional distribution of RA-responsive GCs in the mouse hippocampus and characterizes their properties. Interestingly, we observed that RA depletion stimulates GC activation, leading to an increased number of activated GCs, which indicates a suppressive role of RA in scaling down GC activation evoked by a NE. Moreover, we demonstrate that chronic dietary RA deficiency impairs spatial discrimination in behavioral assays using the Intellicage™ system. Our findings shed light on RA’s integral role in modulating the GC activity in the adult hippocampus and its impact on memory precision.

## 2 Materials and methods

### 2.1 Animals

Wild-type C57BL/6J mice (DBL, Republic of Korea) were utilized at 5 weeks of age at the onset of the experiments. The animals were maintained in group housing conditions, with 4–5 per cage, under a 12-h light/dark cycle at room temperature. They had access to a standard diet and water *ad libitum*. RARE-LacZ mice (TG(RARE-Hspa1b/lacZ)12Jrt/J, JAX stock #008477) were bred to maintain hemizygosity (Rossant et al., 1991). These transgenic mice were between 8 and 15 weeks old at the start of the experiments. All experimental procedures were approved by the DGIST Animal Care and Use Committee, Republic of Korea (DGIST-IACUC-19040202-0006).

### 2.2 Dietary depletion of vitamin A

Dietary treatment commenced when the C57BL/6J mice reached 5 weeks of age. The mice were housed in groups of 4–5 per cage and were provided with either a vitamin A deficient (VAD) diet (TD.86143, Envigo) or a control diet (TD.91280, Envigo). Body weight and pellet consumption for all mice were recorded every week. Behavioral assessments were initiated after 12 weeks on the VAD diet. Subsequently, vitamin A replenishment began with the introduction of a standard diet for 3 weeks.

### 2.3 Stereotaxic infusion of retinoic acid receptor beta antagonist LE135 and its electrophysiological recording

To prepare the LE135 solution, 10 mg of LE135 was dissolved in 2.3 ml DMSO to make a 10 mM stock solution. On the injection day, LE135 was diluted with saline at 10 μM. Stereotaxic infusion of retinoic acid receptor beta (RARβ) antagonist LE135 was performed using an Angle Two™ stereotaxic frame (Leica, Grove, IL, USA). Primary anesthesia was induced by adding 5% isoflurane to the chamber, and 1%–3% isoflurane was given to anesthetized mice using a nose mask with a stereotaxic instrument. Depending on the drug group, each mouse was delivered vehicle (0.1% DMSO in saline) or LE135 500 nl per injection site into bilateral dorsal DG region (AP: −2.00; ML: ±1.35; DV: −1.95) using a nanofil syringe. The flow rate was controlled at 100 nl/min by a Legato^®^ 130 controller (KD Scientific, USA). Following the injection, 5 min waiting period was observed before pulling out the needle and closing the incision; then, the mice were returned to their home cage for recovery. Mice were given a 3-day rest before proceeding with the electrophysiological recording experiments.

Acute hippocampal slices (thickness, 300 μm) were prepared from the brains of C57BL/6J mice of either sex. Mice were anesthetized with isoflurane and decapitated immediately. All samples were obtained coronally for the dorsal hippocampus. Slices were prepared in an oxygenated ice-cold physiological saline using a vibratome (VT1200S, Leica), incubated at ∼32°C for 30 min, and subsequently maintained in the artificial cerebral spinal fluid (ACSF) at room temperature until the recordings. Recordings were performed at near-physiological temperature (∼32°C) in an oxygenated ACSF.

Patch pipettes were obtained from borosilicate glass capillaries (outer diameter = 1.5 mm, inner diameter = 1.1 mm) with a horizontal pipette puller (P-1000, Sutter Instruments). The open-tip resistance of patch pipettes was 4.5–6.5 MΩ for recordings. Current clamp recordings were performed with an EPC-10 USB Double amplifier (HEKA Elektronik) and stored using Patchmaster software. In current-clamp recordings, series resistance was 10–20 MΩ. All experiments were performed on visually identified GCs under DIC optics. GCs located in the middle regions of the suprablade GC were purposely targeted. Around 3 min after patch break-in, resting membrane potential (RMP) was measured, and input resistance (Rin) was determined by applying Ohm’s law to the steady-state voltage difference resulting from a hyperpolarizing current (−10 pA, 500 ms). Pipette capacitance and series resistance (Rs) compensation (bridge balance) were done at the beginning of the clamp recordings.

The extracellular solution for dissection was a choline chloride-based solution (25 mM NaHCO_3_, 2.5 mM KCl, 1.25 mM NaH_2_PO_4_, 7 mM MgCl_2_, 0.5 mM CaCl_2_, 25 mM glucose, 11.61 mM ascorbic acid, 3 mM pyruvic acid, and 110 mM choline chloride). Physiological saline for experiments was standard ACSF (119 mM NaCl, 26 mM NaHCO_3_, 2.5 mM KCl, 1.25 mM NaH_2_PO_4_, 1 mM MgSO_4_, 2 mM CaCl_2_, 0.4 mM ascorbic acid, 2 mM pyruvic acid, and 20 mM glucose). For whole-cell recording, we used K^+^ rich intracellular solution that contained 125 mM K-gluconate, 20 mM KCl, 10 mM HEPES, 0.5 mM EGTA, 4 mM ATP, 10 mM phosphocreatine, and 0.3 mM Tris GTP, pH adjusted to 7.2–3 with KOH (∼300 mOsm).

### 2.4 Tissue preparation and immunohistochemistry

All mice were anesthetized with Avertin (250 mg/kg) via intraperitoneal injection and perfused with PBS, followed by 4% paraformaldehyde (PFA). The brains were then extracted and post-fixed in 4% PFA overnight, dehydrated in 15% sucrose for 12 h, and finally in 30% sucrose overnight at 4°C. The fully saturated brains were sectioned coronally at 40 μm using a Cryostat (CM3050S, Leica). The sections underwent a free-floating treatment process.

For immunostaining, sections were first blocked with blocking buffer (5% goat serum in PBS) for 1 h at room temperature (RT). They were then incubated with primary antibodies diluted in the blocking buffer for 24 h at 4°C. The primary antibodies used included β-galactosidase (chicken, polyclonal IgY, 1:1,000, ab9361, Abcam), calbindin1 (mouse, monoclonal IgG1, 1:750, CB300, Swant), and c-Fos (rabbit, polyclonal, 1:500, ab102499, Abcam), Prox1 (rabbit, polyclonal, 1:500, #925201, BioLegend), Parvalbumin (mouse, monoclonal, 1:250, PV235, Swant). After incubation, sections were washed thrice with washing buffer (0.2% Triton X-100 in PBS) for 5 min each at RT. Sections were then incubated with Alexa Fluor-conjugated secondary antibodies (1:400, Life Technologies) or DRAQ5 (1:2,000, 62251, ThermoFisher) for 3 h at RT. Following this, sections were rewashed thrice with washing buffer and stained with DAPI (1:1,000, Sigma-Aldrich). Finally, they were mounted with Prolong Gold (Life Technologies, USA) anti-fade mounting medium. Imaging and analysis of the sections were performed using a Zeiss LSM800 confocal microscope. Fluorescence-positive cells were quantified through hand-counting and using MetaMorph software, and regions of interest (ROIs) within the DG were evaluated for their area size. The cell count was normalized by the area size of the ROI, and the cell density is presented as [counting number per mm^2^].

### 2.5 Quantitative real-time PCR

To monitor RA depletion in the VAD model, livers were harvested post-euthanasia under deep anesthesia. Each liver was rapidly sectioned into eighths at 4°C and immediately flash-frozen in liquid nitrogen. Total RNA was extracted from the liver sections using the Axygen^®^ AxyPrep MicroRNA (miRNA) Miniprep Kit and subsequently reverse-transcribed into cDNA. Quantitative PCR (qPCR) was performed using 10 ng of cDNA with the following primers for RARβ: 5′-TATGAGATGACAGCGGAGCTAGAC-3′ (forward) and 5′-GGCTTTCCGGATCTTCTCAGT-3′ (reverse). Each sample was assayed in triplicate. The qPCR analysis was conducted on an AriaMx Real-Time PCR system (Agilent Technologies, CA, USA) using the following thermal cycling conditions: an initial denaturation at 95°C for 30 s, followed by 40 cycles of denaturation at 95°C for 5 s and annealing/extension at 60°C for 30 s (Basu et al., 2015). The relative quantification of mRNA levels was determined, including normalization to the gapdh housekeeping gene.

### 2.6 Environmental novelty-evoked GC activation

Environmental novelty-evoked GC activation was assessed after acclimating all mice in the test room for a sufficient period. The NE consisted of a box (30 cm × 30 cm × 30 cm) outfitted with toys serving as novel objects. As indicated in the figures, each mouse was introduced into the box and kept there for the duration specified in each figure. Subsequently, the mice were quickly perfused, and immunohistochemistry was performed with c-Fos staining to identify activated GCs. The control group remained in their home cages until the time of perfusion. To assess c-Fos expression in RA-responsive GCs, animals were exposed to NE for 1.5 h, coinciding with peak expression of immediately early genes as a neural activation marker in active neurons (Chaudhuri et al., 2000; Paul et al., 2020). Additionally, a 30-min NE exposure was used to examine the effect of VAD on GC activation at the time point when GC activation is on the rise but has not yet reached its peak.

### 2.7 Behavioral testing

General behavior tests in the Phenotyper™: Cages were acclimated to the test room for 1 h during a light cycle. Each mouse was placed in a Phenotyper™ (Noldus, USA) enclosure for 24 h, equipped with a feed tray, a water bottle, and an overhead camera. Locomotion and feeding frequency were monitored using EthoVision™ software (Noldus, USA) (Jankovic et al., 2019; Rhine et al., 2019).

#### 2.7.1 Anxiety-based tests (OFT, L&D, and EPM)

Mice were brought to the test room 1 h prior to testing. For the open field test (OFT), each mouse was introduced into an OF box (30 cm × 30 cm × 30 cm), and locomotion was tracked for 30 min (Nestler et al., 2002). On the following day, mice were placed in a light and dark (L&D) box of the same dimensions. Locomotion and time spent in the light zone were recorded for 10 min from when the door was opened (Nestler et al., 2002). On the last day, each mouse was positioned in the center of an elevated plus maze (EPM), facing a closed arm (30 cm in length, 5 cm in width, and elevated 50 cm off the ground). The time spent in each arm and overall locomotion were measured (Duman et al., 2016). All behaviors were observed and quantified using EthoVision™ software to record and analyze the time spent in each designated area.

#### 2.7.2 Spatial discrimination test in Intellicage™

One week prior to testing, a radio frequency identification (RFID) chip was implanted subcutaneously at the nape of each mouse using an injector. Each group of mice was then introduced to the Intellicage™ enclosure (TSE Systems, 20 cm × 55 cm × 38 cm) (Galsworthy et al., 2005; Holgate et al., 2017; Sun et al., 2021). Feed trays and shelters were centrally located within the enclosure. Each corner was equipped with a door granting access to two water bottles. During the initial 5-day acclimation period, all doors remained open to allow free access to water. For the subsequent 4-day nose-poke adaptation period, doors would automatically open for 5 s in response to a nose poke. During the 2-day place learning period, one corner’s water was replaced with a 4% sucrose solution as a reward, with all doors functioning as they did during the adaptation period. In the final 2-day reversal learning period, the reward location was switched to the diagonally opposite corner. Individual visits and licks at each corner were recorded. Between each phase of the test, all components of the Intellicage™ were cleaned with ethanol. The experimental algorithms used within the Intellicage™ system are detailed in Supplementary Figure 5.

#### 2.7.3 Sucrose preference test (SPT)

Prior to the test, each mouse was placed in a single cage with a sufficient supply of pellets in the test room for 1 h to acclimate. At the onset of the light cycle, two bottles were introduced to each cage: one containing water and the other containing a 4% sucrose solution. After 12 h into the light cycle, the consumption from each bottle was determined by measuring the weight change, and then the positions of the bottles were switched. Following a 12-h dark cycle, the weight change of each bottle was measured once more to assess preference (Bernard and Halpern, 1968).

### 2.8 Data presentation and statistics

Data are expressed as the mean ± standard error of the mean (SEM). Statistical analyses were performed using GraphPad Prism software, Version 8.4. The data were compared using an unpaired, two-tailed *t*-test, two-way ANOVA was employed, with Šídák’s and Tukey’s *post-hoc* test applied for further analysis. The results of all statistical tests are described in Supplementary Table 1. Significance levels are denoted as follows: *p < 0.05, **p < 0.01, ***p < 0.001, ****p < 0.0001, with “ns” indicating no significance.

## 3 Results

### 3.1 RA-responsive subpopulation within the dentate GCs

**FIGURE 1:**
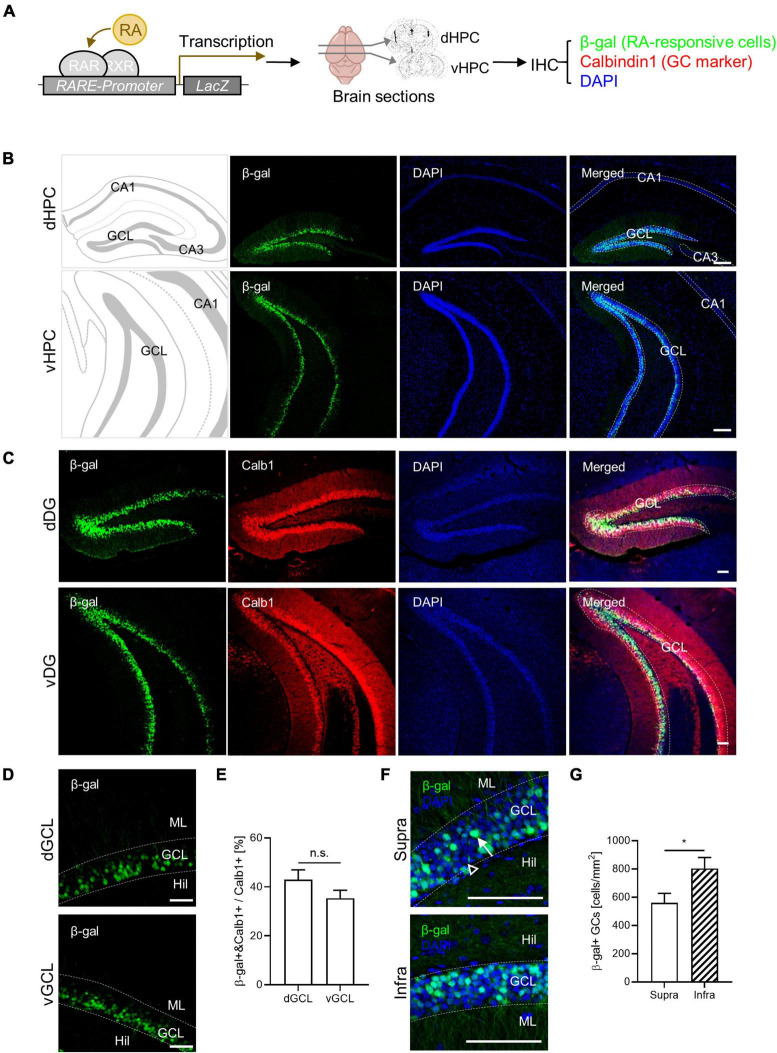
Retinoic acid (RA)-responsive subpopulation exists within the dentate GCs. **(A)** Experimental schematics for β-gal-labeling of RA-responsive cells in the hippocampus. **(B)** Representative images for the spatial distribution of β-gal-positive cells in the dorsoventral axis of the hippocampus. Scale bar, 200 μm. **(C)** Colocalization of β-gal expression with calbindin1, a GC marker, in the dorsal and ventral DG. Scale bar, 100 μm. **(D)** High-resolution images of β-gal-positive cell patterns in the dorsal and the ventral hippocampus. Scale bar, 50 μm. **(E)** Quantitative comparison of β-gal-positive GCs between the dorsal and the ventral hippocampus. (dGCL: *n* = 4, vGCL: *n* = 5 sections). **(F)** High-resolution images of β-gal-positive cell patterns on the suprablade and infrablade of the dorsal DG. Scale bar, 100 μm. **(G)** Quantitative comparison of β-gal-positive cells on the suprablade and the infrablade (Supra: *n* = 10, Infra: *n* = 10 sections). *p < 0.05.

Retinoic acid-responsive cells have been identified in the adult mouse hippocampus (McCaffery et al., 2006; Goodman et al., 2012). In this study, we characterized the spatial distribution of RA-responsive cells in the DG. Utilizing RARE-LacZ reporter mice, we visualized RA-responsive cells where β-galactosidase (β-gal) expression is induced by RARβ binding to RARE (Figure 1A). We found β-gal-positive RA-responsive cells localized to the GC layers along the dorsoventral axis of the DG, but not within the CA1 or CA3 subregions, corroborating previous findings (McCaffery et al., 2006; Goodman et al., 2012). Notably, β-gal expression coincided with the mature GC marker calbindin1 (Calb1) in the DG (Figure 1C). Approximately 40% of the calbindin1-positive mature GCs were RA-responsive (42.9% ± 4.1% in dGCL, 35.4% ± 3.3% in vGCL), a proportion maintained throughout the dorsoventral extent of the hippocampus (Figures 1C–E). RA-responsive cells were more prevalent in the infrablade compared to the suprablade of the DG, revealing an uneven spatial distribution within these subregions (Figures 1F, G). Furthermore, the β-gal signal colocalized with Prox1 an excitatory GC marker, but not with parvalbumin an inhibitory basket cell marker, indicating the cell type identity of RA-responsive cells in the DG (Supplementary Figure 1). Thus, we have delineated a distinct RA-responsive subpopulation among mature GCs and mapped their specific spatial distribution within the DG.

### 3.2 The non-reactive nature of RA-responsive GCs to NE stimulus

**FIGURE 2:**
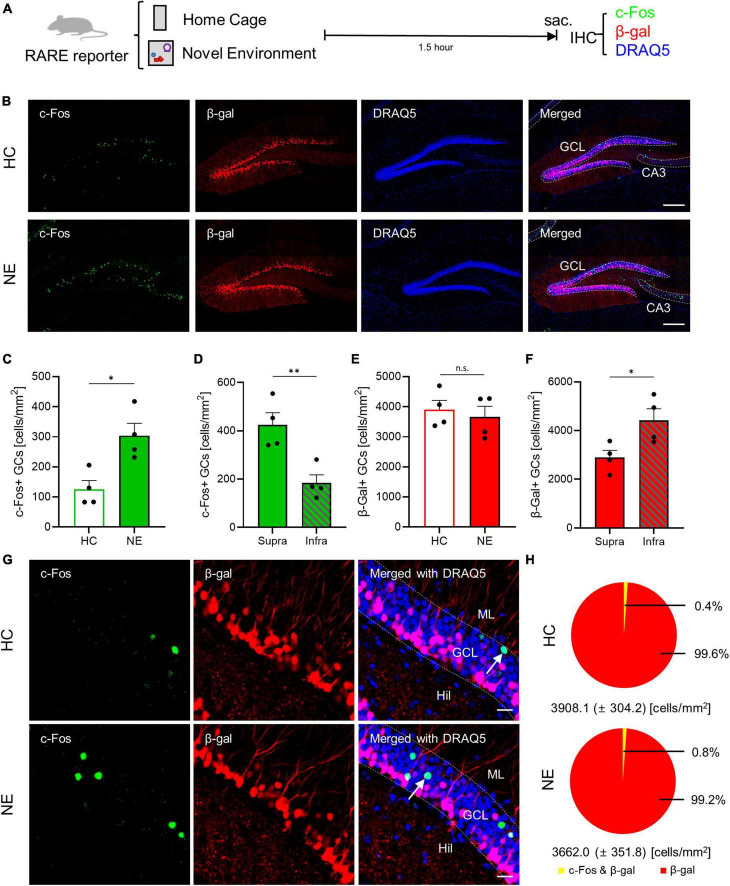
Retinoic acid (RA)-responsive GCs are non-reactive to NE stimuli. **(A)** Experimental schematics for NE-induced GC activation in RARE-LacZ TG mice. **(B)** Representative images of c-Fos expression in RARE-LacZ mice, stained with c-Fos (green), β-gal (red), and DRAQ5 (blue). Scale bar, 200 μm. Quantitative comparison of c-Fos-positive GCs induced by **(C)** HC and NE (HC: *n* = 4, NE: *n* = 4 mice) and **(D)** on suprablade and infrablade in NE (Supra: *n* = 4, Infra: *n* = 4 mice). Quantitative comparison of β-gal-positive GCs induced by **(E)** HC and NE (HC: *n* = 4, NE: *n* = 4 mice), and **(F)** in suprablade and infrablade (Supra: *n* = 4, Infra: *n* = 4 mice). **(G)** High-resolution images of c-Fos, β-gal, and DRAQ5 immunolabeling results. Scale bar, 20 μm. **(H)** Colocalization assay between c-Fos- and β-gal-positive cells within GCs. *p < 0.05, **p < 0.01.

Granule cells in the DGs are known to be sparsely activated in response to spatial or contextual stimuli, a critical feature for the pattern separation process within the DG, which enables discrimination between similar contexts or spaces (Erwin et al., 2020; Lamothe-Molina et al., 2022). RARE-LacZ reporter mice were used to investigate the reactivity of RA-responsive cells to a NE. These mice were either exposed to NE or remained in their home cage (HC) as a control (Figure 2A). For immunohistochemical analysis, β-gal was used as a marker for RA-responsive GCs, and c-Fos served as an immediate early gene indicator of neuronal activation. The density of GCs exhibited no difference between the supra- and infrablade of GC layers (Supplementary Figure 2). Therefore, the area value of each subregion was used to calculate the density of RA-responsive and c-Fos-positive GCs in the supra- and infrablade of the GC layer. Notably, the relative number of β-gal-positive RA-responsive GCs remained constant, showing no change with NE exposure (Figures 2B, E). In contrast, a significant increase in c-Fos-positive activated GCs was observed following NE exposure compared to the HC group (Figures 2B, C). Notably, while RA-responsive GCs were distributed more in the infrablade of the DG than the suprablade (Figures 2B, F), c-Fos-positive GC activation was notably higher in the suprablade than in the infrablade (Figures 2B, D). High-resolution imaging revealed that c-Fos immunoreactivity rarely overlapped with the β-gal signal within the GC layer of the DG (Figures 2G, H). The density of activated GCs due to NE exposure increased from 125.8 ± 29.0 to 304.0 ± 41.0 cells/mm^2^ (Figure 2C). However, the proportion of GCs positive for both c-Fos and β-gal constituted less than 0.8% of the total RARE-positive GCs (Figure 2H), suggesting that RA-responsive GCs are non-reactive to activation by NE stimuli. This finding underscores the specificity of RA signaling pathways in the DG and highlights the potential for distinct regulatory mechanisms governing the activation of RA-responsive GCs in response to environmental changes.

### 3.3 Abnormal increase in NE-evoked GC activation by inhibition of RA signaling

**FIGURE 3:**
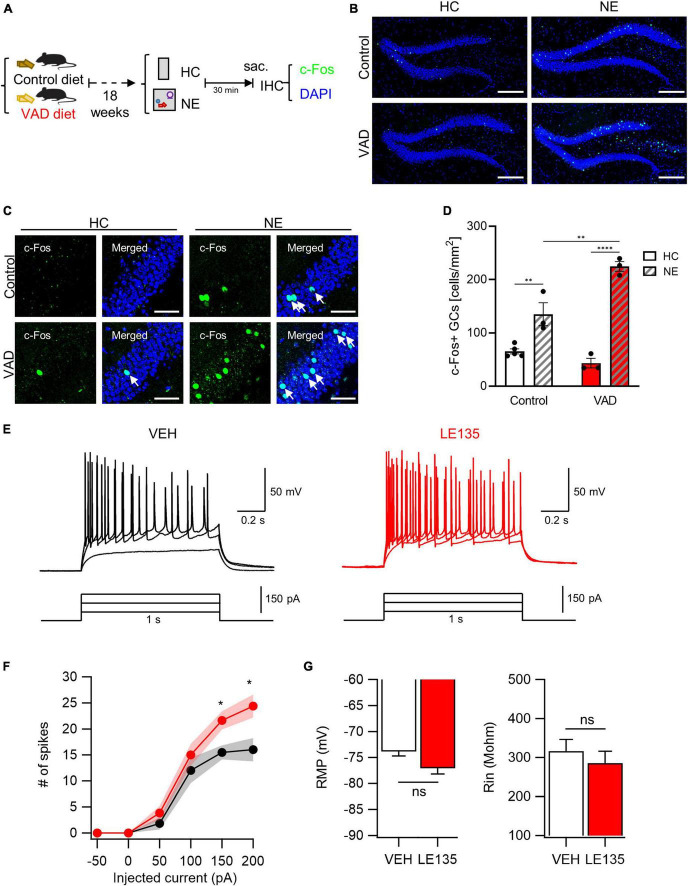
Inhibition of RA signals leads to an abnormal increase in GC activation evoked by NE stimuli. **(A)** Schematics to examine the effect of dietary RA depletion on NE-induced GC activation. **(B)** Representative images of c-Fos immunoreactivity with DAPI nuclear staining. Scale bar, 200 μm. **(C)** High-resolution images of the c-Fos-positive cell patterns within GCs. Scale bar, 50 μm. **(D)** Quantitative comparisons of c-Fos-positive cells between control groups and VAD groups (Control-HC: *n* = 5, Control-NE: 3, VAD-HC: *n* = 3, VAD-NE: *n* = 3 mice). **(E)** The representative traces of vehicle (VEH, black) and RARβ antagonist (LE135, red) in response to depolarizing step current injection. **(F)** The firing frequency as a function of injected current amplitude. Black and red shading represents the SEM for each dataset over current. **(G)** Resting membrane potentials (RMP) and input resistance (Rin) are comparable between groups (VEH: *n* = 13, LE135: *n* = 12 cells). *p < 0.05, **p < 0.01, ****p < 0.0001.

Prompted by our initial observations, we explored the potential causal relationship between RA signaling and GC excitability. We first assessed the impact of dietary vitamin A depletion on GC activation. Mice aged 5 weeks were fed either a VAD diet or a control diet for 18 weeks (Etchamendy et al., 2003). There was no observed difference in body weight gain between control and VAD mice (Supplementary Figure 3A). However, a significant reduction in the expression of the autoregulatory gene RARβ, an indicator of RA signaling, was noted in the livers of VAD mice, confirming systemic RA depletion in our model (Supplementary Figure 3B). Subsequent exposure to a NE or maintenance in the HC was followed by an assay for c-Fos immunoreactivity (Figure 3A). Notably, VAD mice exhibited a more significant increase in NE-induced activated GCs than controls (Figure 3B). High-resolution imaging and quantitative analysis further confirmed that chronic dietary RA depletion significantly increases GC activation when exposed to NE (Figures 3C, D).

We then explored the effects of acute pharmacological inhibition of RARβ on GC activation. The RARβ antagonist LE135 (Yin et al., 2014), or vehicle was infused into the bilateral DG. Three days after stereotaxic surgery, we performed whole-cell patch clamp recordings and measured intrinsic properties on acute hippocampal slices. The passive properties, including RMP (VEH, −73.8 ± 0.9 mV; LE135, −77.0 ± 1.1 mV; *p* = 0.060) and input resistance (VEH, 316.4 ± 30.1 MΩ; LE135, 285.2 ± 30.4 MΩ; *p* = 0.496), were not significantly different between groups (Figure 3G). However, GCs in LE135-treated group (LE135) showed higher excitability than GCs in control group (VEH) in response to 1 s depolarization step current pulse (Figures 3E, F), supporting inhibitory effect of RA signaling on GC excitability in the DG. Through chronic dietary vitamin A depletion and acute pharmacological RARβ inhibition approaches, we have demonstrated that RA signaling suppresses RA-responsive GCs, thereby scaling down NE-induced GC activation in the DG. This abnormal increase in GC activation aligns with the observed non-reactive nature of RA-responsive GCs, suggesting a complex interplay between RA signaling and GC responsiveness to environmental stimuli.

### 3.4 Effect of RA modulation on behavioral spatial discrimination

**FIGURE 4:**
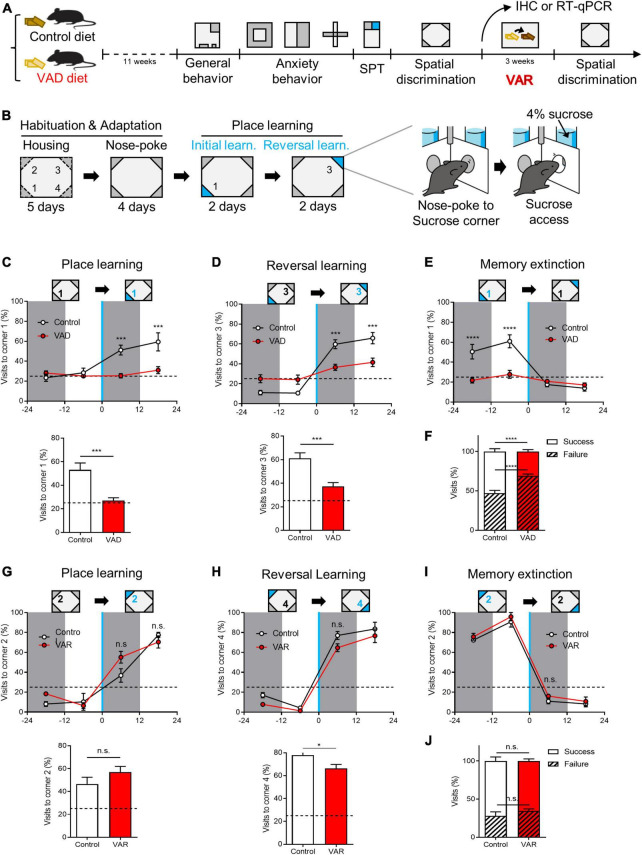
Dietary depletion of RA reversibly impairs spatial discrimination in the Intellicage™ paradigm. **(A)** Schematics for the effect of dietary RA depletion on behavioral phenotypes. **(B)** A behavioral paradigm of spatial discrimination test in the Intellicage™ device. **(C)** Sucrose-corner (corner 1) preference from 1 day before the place learning to the first day of place learning state (above), and the total success rate (below). Dotted line: preference 25%. **(D)** Sucrose-corner (corner 3) preference of reversal learning state (above) and the total success rate (below). Dotted line: preference 25%. **(E)** Sucrose-removed-corner (corner 1) preference of learning state. Dotted line: preference 25%. **(F)** Total visits to sucrose-corner (corner 1) or neutral corners in spatial learning. [**(A–F)** Control: *n* = 10, VAD: *n* = 10]. **(G)** Sucrose-corner preference (corner 2) of place learning state after VA replenishment (above) and the total success rate (below). Dotted line: preference 25%. **(H)** Sucrose-corner preference (corner 4) of reversal learning state. Dotted line: preference 25%. **(I)** Sucrose-removed-corner (corner 2) preference of learning state. Dotted line: preference 25%. **(J)** Total visits to sucrose-corner (corner 2) or neutral corners in spatial learning after VA replenishment. [**(G–J)** Control: *n* = 4, VAD: *n* = 4]. *p < 0.05, ***p < 0.001, ****p < 0.0001.

Building on our discovery of RA’s role in modulating GC activation within the DG, we investigated the impact of RA depletion on hippocampus-dependent behaviors. Control and VAD mice were subjected to 18 weeks of dietary treatment before undergoing a battery of behavioral tests to assess general activity, anxiety, and spatial learning (Figure 4A). VAD did not significantly affect food consumption or voluntary movement in the general behavior tests (Supplementary Figures 4A, B) nor altered anxiety-related behaviors in the OFT, L&D, and EPM tests (Supplementary Figures 4C–E). Similarly, sucrose preference remained unchanged between VAD and control mice (Supplementary Figures 4F–H).

Spatial discrimination was evaluated using the Intellicage™ system and a place learning paradigm heavily reliant on hippocampal function (Galsworthy et al., 2005; Maroteaux et al., 2018; Voikar et al., 2018). The test involved distinguishing between identical chambers at the four corners of the cage, with access to water contingent upon visiting and nose-poking specific chambers (Supplementary Figures 5A–D). Each mouse was equipped with a RFID tag to monitor corner visits. Following habituation and adaptation to the Intellicage™, mice underwent place learning sessions, including initial learning and reversal learning phases. Corner preferences were measured before and after introducing sweetened water (4% sucrose) at a single corner (Figure 4B). Control mice gradually increased visits to the sucrose-designated corner during the initial learning session, whereas VAD mice demonstrated significantly poorer performance in locating the sucrose corner (Figure 4C). This pattern persisted during the reversal learning session, which evaluated the acquisition of new spatial memories (corner 3) and the extinction of previous ones (corner 1) (Figures 4D, E). Overall, control mice achieved a 52.8% ± 3.5% success rate in learning tasks, while VAD mice managed only a 31.1% ± 2.5% success rate (Figure 4F), suggesting that RA depletion via a VAD diet impairs spatial discrimination abilities in adult mice.

To determine if impaired spatial discrimination could be reversed, VAD mice were switched to a control diet immediately after the initial spatial discrimination test, creating a Dietary vitamin A replenishment (VAR) model (Bonhomme et al., 2014) over 3 weeks in their HC (Figure 4A). Subsequently, both control and VAR mice were retested for spatial discrimination. During the initial learning session, VAR mice displayed a complete restoration of their ability to locate the sucrose corner (corner 2), performing on par with control mice (Figure 4G). Similarly, during reversal learning, VAR mice successfully shifted their preference from the previously rewarded corner (corner 2) to the new sucrose corner (corner 4), mirroring the adaptability of control mice (Figures 4H, I). Overall, both groups showed comparable success rates in the total learning period, with control mice at 71.9% ± 5.3% and VAR mice at 65.7% ± 2.8% (Figure 4J). These results underscore the vital role of RA, the bioactive derivative of vitamin A, in hippocampus-dependent spatial discrimination.

## 4 Discussion

The role of RA in regulating synaptic plasticity is well established (Chen et al., 2014; Arendt et al., 2015; Zhong et al., 2018; Lenz et al., 2021b), and its homeostatic imbalance, along with disruptions in downstream signaling pathways, has been implicated in a variety of cognitive and affective disorders. These disorders include age-related cognitive decline, Alzheimer’s disease, and major depression (Ding et al., 2008; Soden and Chen, 2010; Bremner et al., 2011; Zhong et al., 2018; Park et al., 2021). Understanding the impact of RA signaling on neuronal circuits is essential for elucidating its physiological and pathological roles in both healthy and diseased states. This study demonstrates that an RA-responsive subpopulation exists within the dentate GCs in the hippocampus (Figure 1). Intriguingly, RA-responsive GCs usually are non-reactive to environmental novelty, and thus remain quiescent (Figure 2). However, chronic dietary RA depletion leads to their increased activation, a response also seen with acute pharmacological RARβ inhibition in the DG (Figure 3). Furthermore, RA depletion impairs, while its replenishment restores, spatial discrimination abilities in mice. These findings provide insight into the role of RA in DG neural circuitry and spatial memory function within the adult hippocampus (Figure 4).

Here, we profiled sub-regional and cell-type distribution of RA-responsiveness within the adult mouse hippocampus (Figure 1). By utilizing a RARE-driven LacZ reporter to indicate β-gal expression, we observed significant labeling in the DG, but not in CA subregions. Furthermore, RA-responsive cells were more abundant in the infrablade than in the suprablade of the DG GC layers. Specifically, our findings suggest that approximately 40% of total calbindin1-positive mature GCs exhibit RA-responsiveness (Figure 1E). Furthermore, our data indicate that inhibitory basket cells do not exhibit detectable β-gal expression, implying cell-type specificity in RA responsiveness among DG cell types. A previous study by Mishra et al. reported β-gal signal in a small subset of neural stem cells, progenitor cells, and neuroblasts during adult neurogenesis, indicating the complexity of RA signaling in the adult hippocampus (Mishra et al., 2018). Although these cell types exhibit a lower level of RA responsiveness compared to mature GCs, the potential implications of these minor RA-responsive populations under neurogenic process should be further characterized, particularly in the context of cognitive functions, including spatial memory.

We found these RA-responsive GCs appeared to be non-reactive to NE stimuli (Figure 2). Interestingly, we noted a higher density of activated GCs in the suprablade than the infrablade (Figure 2D), which starkly contrasts the distribution pattern of RA-responsive GCs across these DG subregions (Figure 1G). Additionally, our results showed that the induction of the neuronal activation marker c-Fos was almost exclusively associated with β-gal signaling within the GC layer of the DG (Figure 2G), with minimal overlap between RA-responsive GCs and c-Fos-positive GCs (Figure 2H). This underscores the resistance of RA-responsive GCs to NE-induced activation. Significantly, we found that chronic RA depletion resulted in an increased number of NE-activated GCs (Figures 3A–D), a phenomenon similarly observed after acute pharmacological inhibition of RARβ within the DG (Figures 3E–G). These observations suggest that RA signaling serves to inhibit the activation of RA-responsive GCs, thus modulating the extent of GC activation in response to NE challenges.

Previous research has indicated that RA can have varying effects on neuronal activity, which seem to be dependent on the specific brain regions and cell types involved (Jiang et al., 2012; Yee and Chen, 2016; Zhong et al., 2018; Hsu et al., 2019; Lenz et al., 2021a). For example, Zhong et al. (2018) demonstrated that RA signaling reduced inhibitory neurotransmission in the visual cortex without affecting excitatory transmission. Conversely, Yee and Chen (2016) found an enhancement in inhibitory synaptic strength with no alteration in excitatory transmission in the somatosensory cortex. Additionally, Lenz et al. (2021a) described the immediate effects of RA on synaptic plasticity in GCs within the mouse DG, noting increased spontaneous excitatory postsynaptic current (sEPSC) frequencies and synapse numbers after RA administration, without changes in sEPSC amplitudes. These disparate results underscore the complexity of RA’s influence on neuronal activation, which appears to be region- and cell-type-specific. It is also important to account for methodological variances across studies, such as the experimental setup (*in vivo* versus *ex vivo*) and the approaches used to manipulate RA levels (varying concentrations, acute versus chronic alterations, and methods of RA administration or depletion) (Enderlin et al., 2000; Misner et al., 2001; Cocco et al., 2002; Aoto et al., 2008; Jiang et al., 2012; Hsu et al., 2019; Huang and Chen, 2020; Lenz et al., 2021a). Our current study adds to this body of knowledge by showing that neuronal activation in GCs evoked by a NE is consistently increased following chronic RA depletion *in vivo*. Our electrophysiological recording revealed that GC firing frequency is notably increased after acute pharmacological inhibition of RARβ in the DG (Figure 3). This suggests that RA typically acts to limit the activation of DG GCs. Looking ahead, research should focus on pinpointing the specific factors regulated by RA signaling within particular neuronal populations and at precise times. Furthermore, it is essential to dissect the transcriptional and non-transcriptional pathways through which RA influences GC neuronal activity in the DG.

The DG, serving as the gateway to the hippocampus, mediates pattern separation, which creates distinct representations of contexts to facilitate memory precision (Leutgeb et al., 2007; McHugh et al., 2007; Yassa and Stark, 2011; Nakashiba et al., 2012). GCs achieve this through sparse activation, encoding contextual information incoming from the entorhinal cortex (EC) (Leutgeb et al., 2007; McHugh et al., 2007; Cayco-Gajic and Silver, 2019). Such sparse yet patterned activation of GCs is fundamental to the unique representation of a given context, thereby discriminating it from similar contexts to enhance memory precision (Yassa and Stark, 2011; Hainmueller and Bartos, 2020). This specificity of GC activation for memory ensembles is tightly regulated by dedicated control of the excitatory/inhibitory balance (Hainmueller and Bartos, 2020). Conversely, excessive interference between memory ensembles can deteriorate memory precision (Besnard and Sahay, 2016; Lange et al., 2017). Elevated uncorrelated activity within GC ensembles may lead to considerable overlap between similar contexts, impairing contextual discrimination. Overactivation of GCs can thus detrimentally affect contextual and spatial information resolution, aligning with previous studies showing that excessive GC activity in epilepsy disrupts pattern separation (Sparks et al., 2020). Our study revealed that RA modulation of RA-responsive GCs influences the precise scaling of GC activation (Figure 3). We also found that an RA-deficient diet increased the number of activated GCs in response to a NE stimulus. This suggests that RA deficiency may prompt aberrant activation of typically dormant RA-responsive GCs (Figure 4). This implies that RA in the DG is instrumental in spatial learning by inhibiting these cells’ responses. These findings raise the possibility that RA contributes to spatial information encoding by minimizing GC ensemble overlap, thereby enhancing spatial discrimination. Future research employing *in vivo* calcium imaging of DG-GCs is warranted to observe neuronal ensembles as mice freely navigate specific spaces.

Granule cells comprise heterogeneous subpopulations characterized by distinct gene expression profiles and electrophysiological properties (Shridhar et al., 2022). The formation of memory ensembles is governed not only by selective activation but also by the proper inhibition of specific GC subpopulations (Guo et al., 2018). Aberrant activation of RA-responsive GCs appears to play an inhibitory role in forming memory ensembles, as blocking RA signaling leads to the overactivation of GCs in response to spatially novel stimuli (Figure 3). This indicates that the RA-responsive GC subpopulation is a crucial element of GC heterogeneity, contributing to the formation of spatial memory engrams. Maintaining the RA-responsive cell population is essential for the development of appropriate neuronal ensembles and behavior because RA-responsive GCs exhibit low neuronal excitability at the individual cell level (Huang and Chen, 2020).

## 5 Conclusion

In conclusion, our research has shed light on the significant role of RA in modulating the GC activity within the DG, a key region implicated in the encoding of spatial information. We have established that RA-responsive GCs form a critical subpopulation that contributes to the broader heterogeneity of GCs. Aberrant activation of these RA-responsive GCs, as seen with RA signaling inhibition, leads to overactivation and may result in impaired spatial discrimination. In addition, this study delineates the impact of RA on DG function but also contributes to our understanding of cognitive processes at a cellular level. It provides a valuable framework for exploring how disruptions in RA signaling could underlie cognitive deficits observed in various brain disorders, including major depression, schizophrenia, epilepsy, and Alzheimer’s disease. By demonstrating that RA signaling is integral to restraining DG-GC activation, thereby enhancing spatial memory precision, our work underscores the potential for targeting RA pathways in therapeutic interventions aimed at mitigating cognitive decline associated with RA dysregulation.

## Data availability statement

The original contributions presented in this study are included in the article/Supplementary material, further inquiries can be directed to the corresponding author.

## Ethics statement

All experimental procedures were approved by the DGIST Animal Care and Use Committee, Republic of Korea (DGIST-IACUC-19040202-0006). The study was conducted in accordance with the local legislation and institutional requirements.

## Author contributions

Y-GY: Conceptualization, Data curation, Investigation, Methodology, Validation, Writing – original draft, Writing – review & editing. JP: Conceptualization, Investigation, Methodology, Supervision, Validation, Writing – review & editing. YK: Methodology, Validation, Visualization, Investigation, Writing – review & editing. J-CR: Methodology, Project administration, Supervision, Validation, Investigation, Writing – review & editing. C-HS: Investigation, Methodology, Writing – review & editing. S-JO: Investigation, Supervision, Writing – review & editing. J-HJ: Investigation, Supervision, Writing – review & editing. YL: Investigation, Writing – review & editing. JY: Funding acquisition, Methodology, Investigation, Writing – review & editing. Y-SO: Conceptualization, Funding acquisition, Project administration, Supervision, Writing – original draft, Writing – review & editing.
