# Supplementary material for: Retinoic acid modulation of granule cell activity and spatial discrimination in the adult hippocampus
Source: Front Cell Neurosci. 2024 Apr 17;18:1379438. doi: 10.3389/fncel.2024.1379438 (PMC11061364; doi:10.3389/fncel.2024.1379438)
Supplement: Supplementary file 1 [file Data_Sheet_1.docx]

Supplementary Material

# Supplementary Figures and Tables

## Supplementary Figures

**
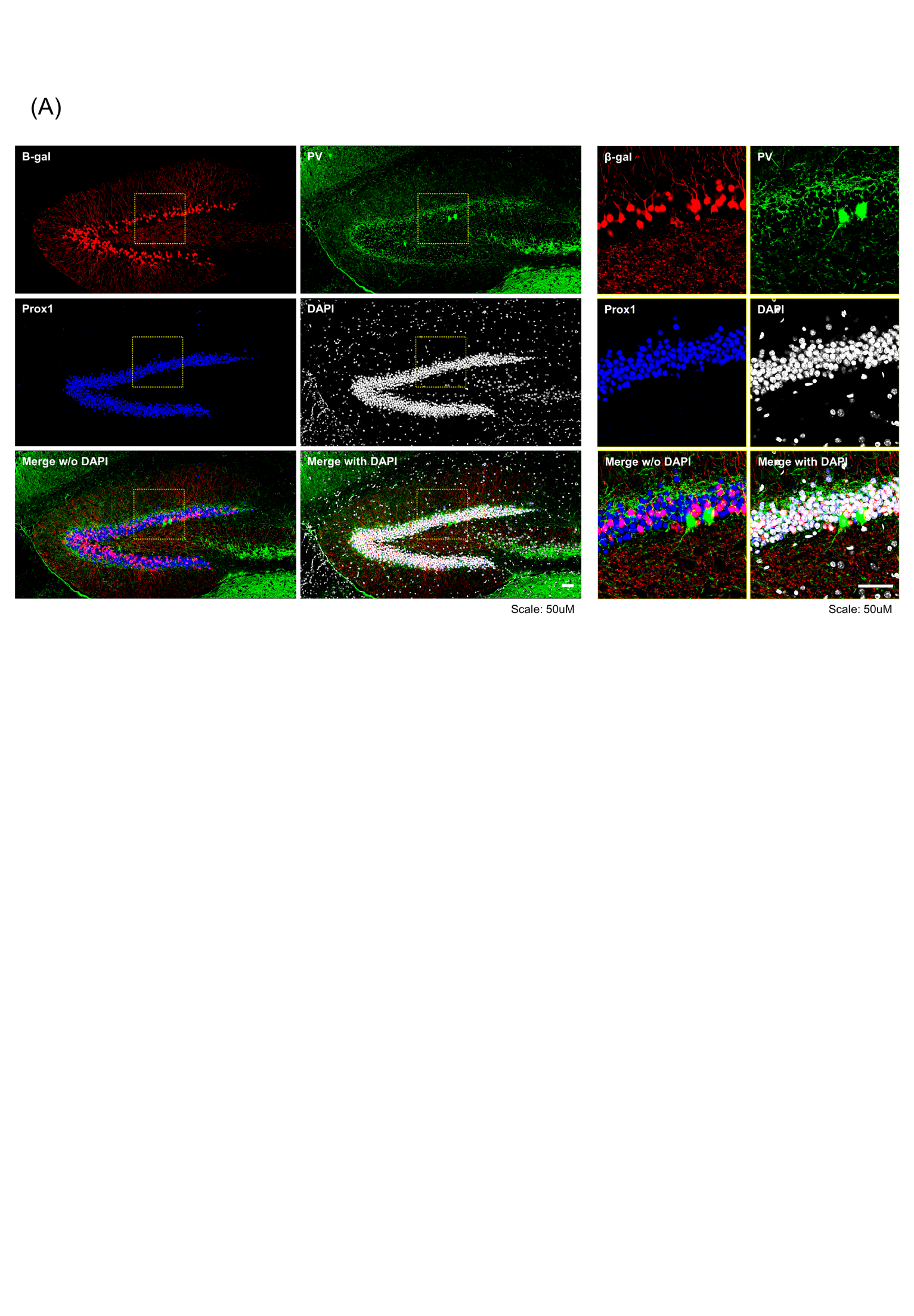
**

**Supplementary Figure 1.** Characteristics of retinoic acid response element (RARE)-positive cells in the dentate gyrus

(A) Representative images of immunohistochemistry results in RARE-LacZ mice. The sections were stained with β-gal (red), PV (Parvalbumin, green), Prox1 (blue), and DAPI (white). Scale bar, 50µm.


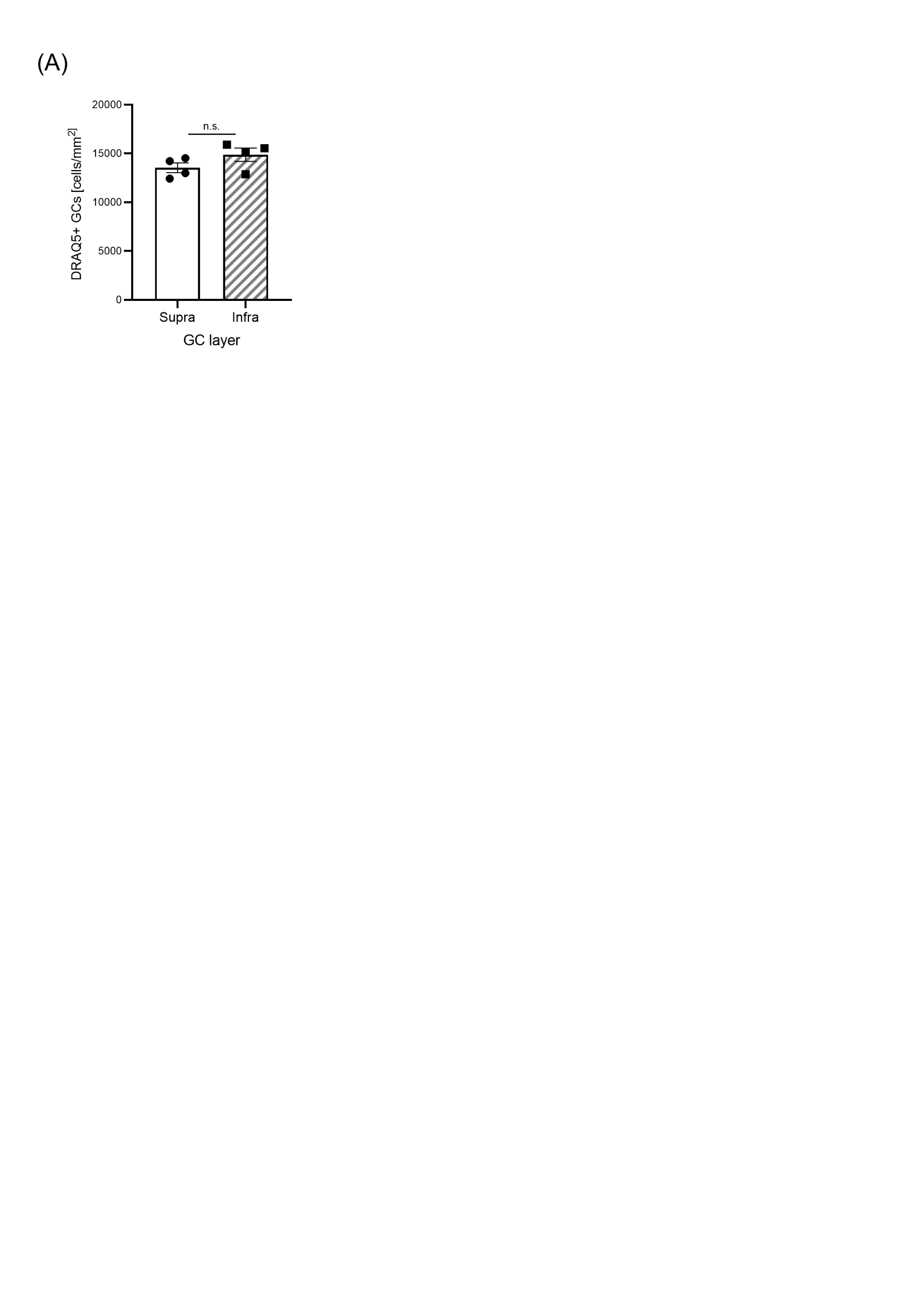


**Supplementary Figure 2.** Relative density of GCs in the supra- and infrablade of GC layer

(A) DRAQ5-positive GC counts normalized with area of the supra- and infrablade of GC layer. (Supra: n = 4, Infra: n = 4 mice)

##
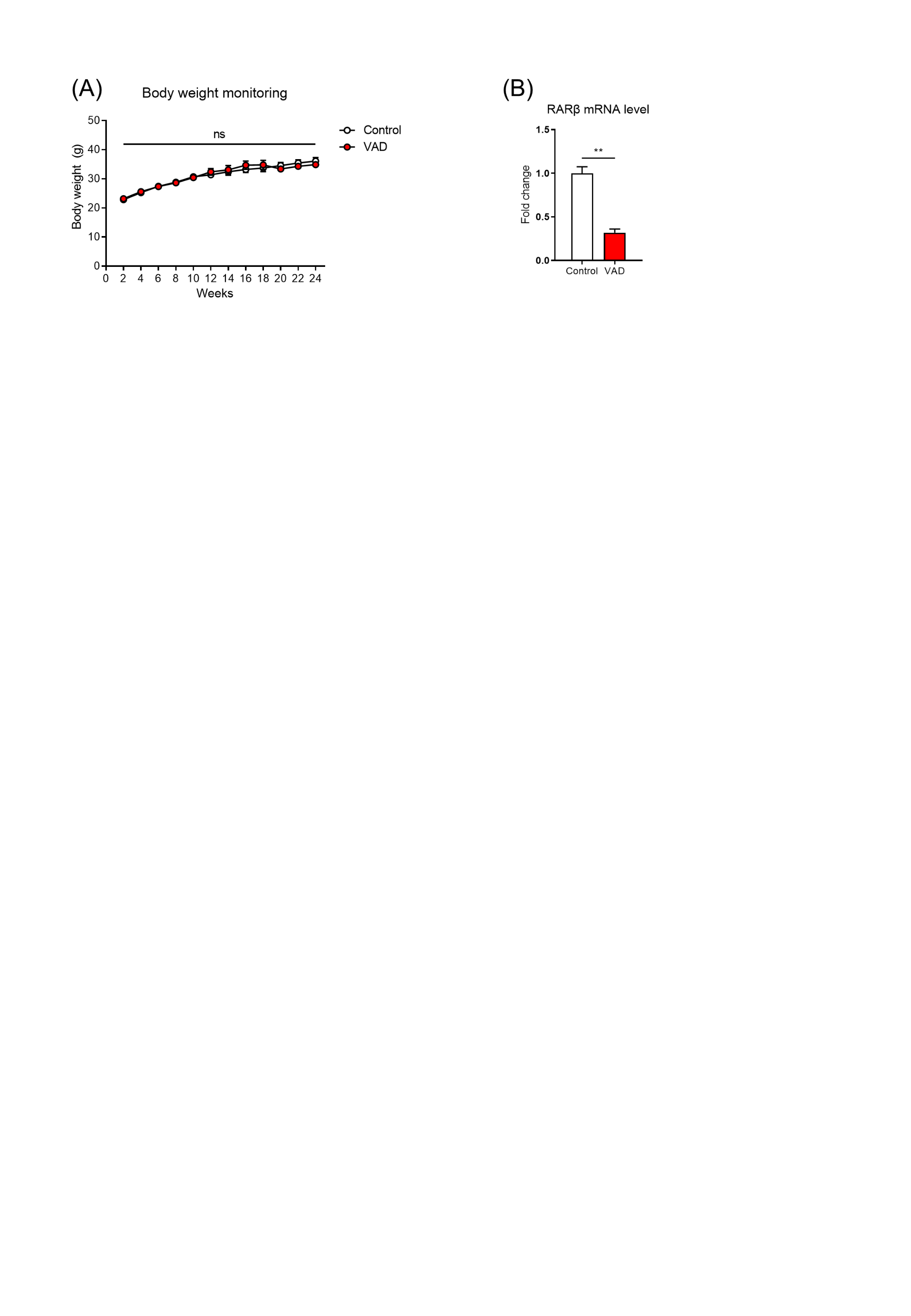


**Supplementary Figure 3.** VAD does not influence body weight

(A) Body weight monitoring every two weeks since VAD diet treatment. (Control: n = 17, VAD: n = 17 mice) (B) Relative RARβ mRNA levels compared to β-actin in the liver of the VAD mice. (Control: n = 4, VAD: n = 7 mice)


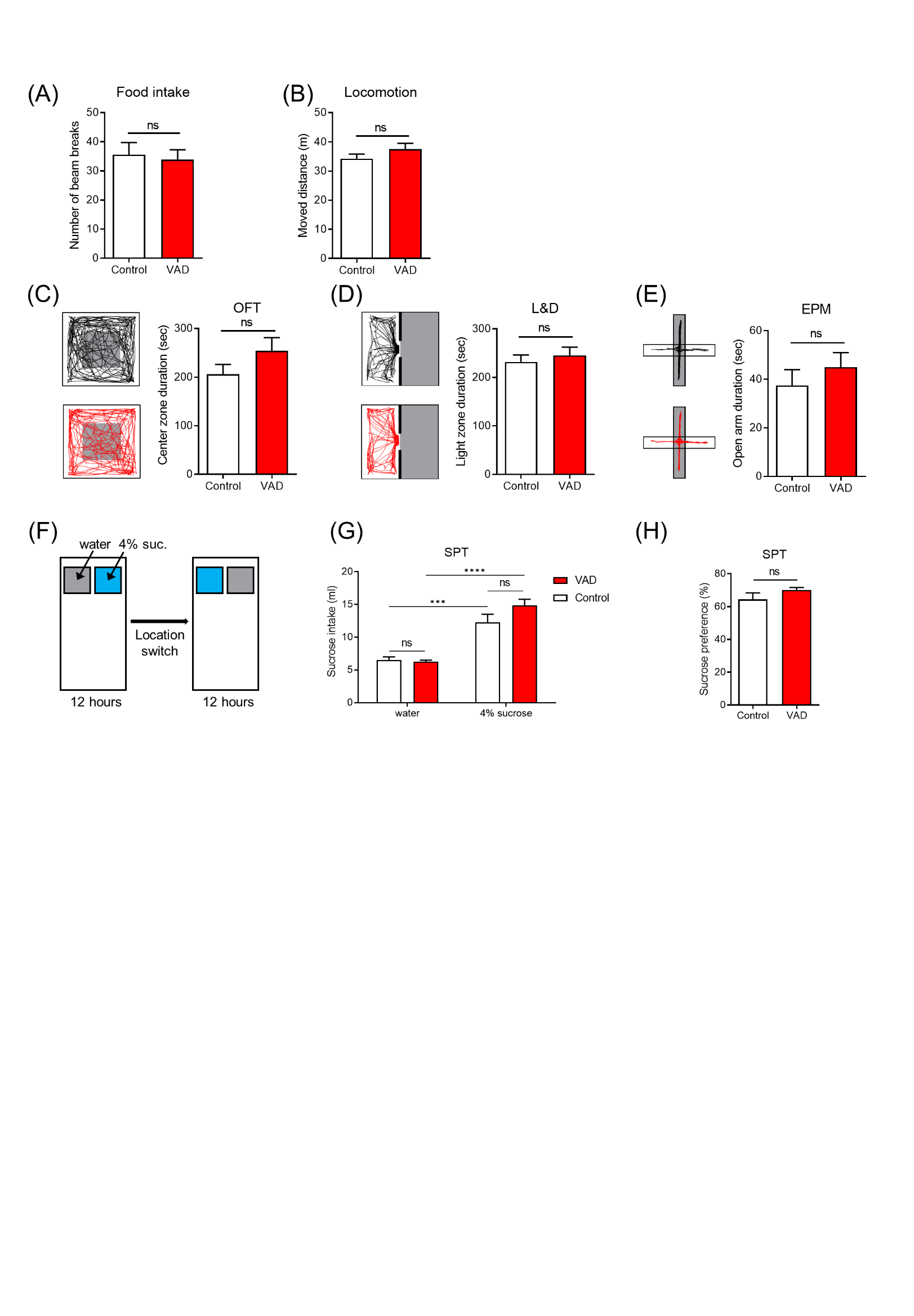


**Supplementary Figure 4.** VAD does not influence general behaviors

(A, B) Average (A) number of accesses to pellets (Control: n = 7, VAD: n = 7 mice) and (B) distance moved in the Phenotyper^TM^ per hour. (Control: n = 17, VAD: n = 17 mice) (C) Representative behaviors of the control mouse (black) and VAD mouse (red) in the open field test. And the average duration in the center zone (gray filled box). (Control: n = 17, VAD: n = 17 mice) (D) Representative behaviors of the control mouse (black) and VAD mouse (red) in the light & darkness box test. And the average duration in the light zone (white-filled box). (Control: n = 17, VAD: n = 17 mice) (E) Representative behaviors of the control mouse (black) and VAD mouse (red) in the elevated plus maze test. And the average duration in the open arms (white-filled boxes). (Control: n = 17, VAD: n = 17 mice) (F) Schematic of sucrose preference test. (G) Intake volume of water or 4% sucrose. (Control: n = 7, VAD: n = 7 mice) (H) Total sucrose preference. (Control: n = 7, VAD: n = 7 mice)


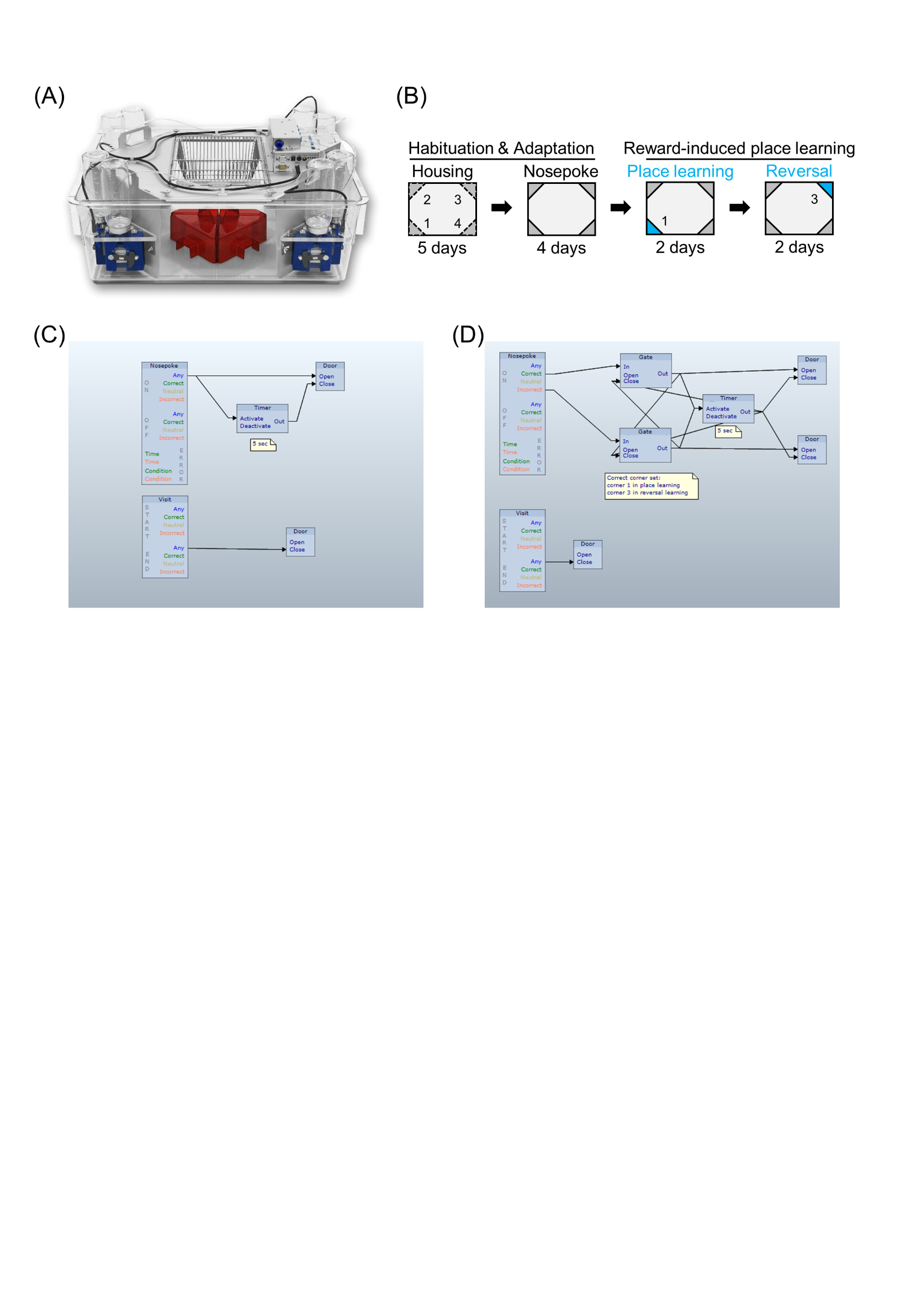


**Supplementary Figure 5.** Experimental algorithms of the Intellicage^TM^ system

(A) Intellicage^TM^ instrument. (B) A behavioral paradigm of spatial discrimination test. (C) System algorithm in place learning period. (D) A system algorithm is in place for the learning and reversal learning periods.

## Supplementary Tables

**Supplementary Table 1.** Statistics

| **Figure** | **Data** | **Test** | **df** | **F-value** | **p-value** | **Factor** | **Groups** | **Numbers** | **Comparison** | **Adj. p-value** | **Significance** |
| --- | --- | --- | --- | --- | --- | --- | --- | --- | --- | --- | --- |
| **Figure 1** |  |  |  |  |  |  |  |  |  |  |  |
| Figure 1E | β-gal+ granule cells (GCs) in dentate gyrus | Two-tailed unpaired t-test | 7 | F(3, 4) = 1.222 | 0.1862 | Dorsoventral axis of dentate gyrus | dGCL, vGCL | Dorsal GCs: 4 Ventral GCs: 5 | dGCL vs. vGCL |  | n.s. |
| Figure 1G | β-gal+ GCs per GC blade | Two-tailed unpaired t-test | 18 | F(9, 9) = 1.307 | 0.0292 | GC blade | Supra, infrablade of GCs | Suprablade GCs: 10 Infrablade GCs: 10 | Suprablade vs. Infrablade |  | * |
| **Figure 2** |  |  |  |  |  |  |  |  |  |  |  |
| Figure 2C | c-Fos+ GCs induction after environmental exposure | Two-tailed unpaired t-test | 6 | F(3, 3) = 2.009 | 0.0121 | Environmental exposure | Homecage (HC), Novel environment (NE) | HC: 4 NE: 4 | HC vs. NE |  | * |
| Figure 2D | c-Fos+ GCs induction per GC blade in NE exposure | Two-tailed unpaired t-test | 6 | F(3, 3) = 2.218 | 0.0075 | GC blade | Supra, infrablade of GCs | Suprablade GCs: 4 Infrablade GCs: 4 | Suprablade vs. Infrablade |  | ** |
| Figure 2E | β-gal+ GCs induction after environmental exposure | Two-tailed unpaired t-test | 6 | F(3, 3) = 1.337 | 0.6157 | Environmental exposure | Homecage (HC), Novel environment (NE) | HC: 4 NE: 4 | HC vs. NE |  | n.s. |
| Figure 2F | β-gal+ GCs induction per GC blade in NE exposure | Two-tailed unpaired t-test | 6 | F(3, 3) = 2.637 | 0.0333 | GC blade | Supra, infrablade of GCs | Suprablade GCs: 4 Infrablade GCs: 4 | Suprablade vs. Infrablade |  | * |
| **Figure 3** |  |  |  |  |  |  |  |  |  |  |  |
| Figure 3D | c-Fos+ GCs induction after environmental exposure in dietary status | Two-way ANOVA and post hoc Tukey’s multiple comparisons tests | Interaction: 1 Environment: 1 Diet: 1 Residual: 10 | 24.81 125.7 9.110 | 0.0006 <0.0001 0.0129 | Environmental exposure, Diet | HC: Control | 5 | HC Control vs. HC VAD | 0.4873 | n.s. |
|  |  |  |  |  |  |  | NE: Control | 3 | NE Control vs. NE VAD | 0.0015 | ** |
|  |  |  |  |  |  |  | HC: VAD | 3 | HC Control vs. NE Control | 0.0041 | ** |
|  |  |  |  |  |  |  | NE:VAD | 3 | HC VAD vs. NE VAD | <0.0001 | **** |
| Figure 3G | F/I curve (# of spikes) | Mann-Whitney U test |  |  | 0.29629 | 50 pA | VEH LE135 | VEH (n=13) LE135 (n=12) | VEH vs. LE135 |  | n.s. |
|  |  |  |  |  | 0.24032 | 100 pA |  |  |  |  | n.s. |
|  |  |  |  |  | 0.01641 | 150 pA |  |  |  |  | * |
|  |  |  |  |  | 0.01776 | 200 pA |  |  |  |  | * |
|  | RMP |  |  |  | 0.06058 |  |  |  |  |  | n.s. |
|  | Rin |  |  |  | 0.49656 |  |  |  |  |  | n.s. |
| **Figure 4** |  |  |  |  |  |  |  |  |  |  |  |
| Figure 4C | Sucrose-corner (corner 1) preference before and after the place learning | Two-way ANOVA and post hoc Sidak’s multiple comparisons tests | 3 3 1 18 54 | F(3, 54) = 11.48 F(3, 54) = 14.93 F(1, 18) = 7.121 F(18, 54) = 4.151 | <0.0001 <0.0001 0.0157 <0.0001 | Diet, time | Time: -18, -6, 6, 18 | 4 time points | Control vs. VAD: -18 | 0.9217 | n.s. |
|  |  |  |  |  |  |  | Diet: Control, VAD | 10 | Control vs. VAD: -6 | 0.9791 | n.s. |
|  |  |  |  |  |  |  |  |  | Control vs. VAD: 6 | 0.0007 | *** |
|  |  |  |  |  |  |  |  |  | Control vs. VAD: 18 | 0.0002 | *** |
| Figure 4C_Column | Sucrose-corner (corner 1) preference before and after the place learning | Two-tailed unpaired t-test | 18 | F(9, 9) = 6.295 | 0.0006 | Diet | Control, VAD | Control: 10 VAD: 10 |  |  | *** |
| Figure 4D | Sucrose-corner (corner 3) preference before and after the reversal learning | Two-way ANOVA and post hoc Sidak’s multiple comparisons tests | 3 3 1 18 54 | F(3, 54) = 17.85 F(3, 54) = 56.60 F(1, 18) = 2.127 F(18, 54) = 1.766 | <0.0001 <0.0001 0.1619 0.0555 | Diet, time | Time: -18, -6, 6, 18 | 4 time points | Control vs. VAD: -18 | 0.0569 | n.s. |
|  |  |  |  |  |  |  | Diet: Control, VAD | 10 | Control vs. VAD: -6 | 0.0699 | n.s. |
|  |  |  |  |  |  |  |  |  | Control vs. VAD: 6 | 0.0004 | *** |
|  |  |  |  |  |  |  |  |  | Control vs. VAD: 18 | 0.0002 | *** |
| Figure 4D_Column | Sucrose-corner (corner 3) preference before and after the reversal learning | Two-tailed unpaired t-test | 18 | F(9, 9) = 2.083 | 0.0006 | Diet | Control, VAD | Control: 10 VAD: 10 |  |  | *** |
| Figure 4E | Sucrose-removed-corner (corner 1) preference of learning state | Two-way ANOVA and post hoc Sidak’s multiple comparisons tests | 3 3 1 18 54 | F(3, 54) = 15.85 F(3, 54) = 30.58 F(1, 18) = 11.59 F(18, 54) = 2.679 | <0.0001 <0.0001 0.0032 0.0027 | Diet, time | Time: -18, -6, 6, 18 | 4 time points | Control vs. VAD: -18 | <0.0001 | **** |
|  |  |  |  |  |  |  | Diet: Control, VAD | 10 | Control vs. VAD: -6 | <0.0001 | **** |
|  |  |  |  |  |  |  |  |  | Control vs. VAD: 6 | 0.9724 | n.s. |
|  |  |  |  |  |  |  |  |  | Control vs. VAD: 18 | 0.9700 | n.s. |
| Figure 4F | Total visits in spatial learning | Two-tailed unpaired t-test | 1 1 1 36 | F(1, 36) = 50.53 F(1, 36) = 0.000 F(1, 36) = 27.99 | <0.0001 >0.9999 <0.0001 | Diet | Control, VAD | Control: 10 VAD: 10 | Control vs. VAD: Success | <0.0001 | **** |
|  |  |  |  |  |  |  |  |  | Control vs. VAD: Failure | <0.0001 | **** |
| Figure 4G | Sucrose-corner preference (corner 2) of place learning state after VA replenishment | Two-way ANOVA and post hoc Sidak’s multiple comparisons tests | 3 3 1 6 18 | F(3, 18) = 3.992 F(3, 18) = 105.0 F(1, 6) = 0.7189 F(6, 18) = 3.051 | 0.0242 <0.0001 0.4290 0.0307 | Diet, time | Time: -18, -6, 6, 18 | 4 time points | Control vs. VAD: -18 | 0.5409 | n.s. |
|  |  |  |  |  |  |  | Diet: Control, VAD | 4 mice each | Control vs. VAD: -6 | 0.9766 | n.s. |
|  |  |  |  |  |  |  |  |  | Control vs. VAD: 6 | 0.0768 | n.s. |
|  |  |  |  |  |  |  |  |  | Control vs. VAD: 18 | 0.8166 | n.s. |
| Figure 4G_Column | Sucrose-corner preference (corner 2) of place learning state after VA replenishment | Two-tailed unpaired t-test | 6 | F(3, 3) = 1.531 | 0.2164 | Diet | Control, VAD | Control: 4 VAD: 4 |  |  | n.s. |
| Figure 4H | Sucrose-corner preference (corner 4) of reversal learning state | Two-way ANOVA and post hoc Sidak’s multiple comparisons tests | 3 3 1 6 18 | F(3, 18) = 0.4872 F(3, 18) = 187.7 F(1, 6) = 7.292 F(6, 18) = 1.000 | 0.6955 <0.0001 0.0356 0.4551 | Diet, time | Time: -18, -6, 6, 18 | 4 time points | Control vs. VAD: -18 | 0.4070 | n.s. |
|  |  |  |  |  |  |  | Diet: Control, VAD | 4 mice each | Control vs. VAD: -6 | 0.9823 | n.s. |
|  |  |  |  |  |  |  |  |  | Control vs. VAD: 6 | 0.162 | n.s. |
|  |  |  |  |  |  |  |  |  | Control vs. VAD: 18 | 0.6796 | n.s. |
| Figure 4H_Column | Sucrose-corner preference (corner 4) of reversal learning state | Two-tailed unpaired t-test | 6 | F(3, 3) = 1.723 | 0.0381 | Diet | Control, VAD | Control: 4 VAD: 4 |  |  | * |
| Figure 4I | Sucrose-removed-corner (corner 2) preference of learning state | Two-way ANOVA and post hoc Sidak’s multiple comparisons tests | 3 3 1 6 18 | F(3, 18) = 0.05817 F(3, 18) = 256.9 F(1, 6) = 3.862 F(6, 18) = 0.5939 | 0.9810 <0.0001 0.0970 0.7313 | Diet, time | Time: -18, -6, 6, 18 | 4 time points | Control vs. VAD: -18 | 0.9426 | n.s. |
|  |  |  |  |  |  |  | Diet: Control, VAD | 4 mice each | Control vs. VAD: -6 | 0.7971 | n.s. |
|  |  |  |  |  |  |  |  |  | Control vs. VAD: 6 | 0.7801 | n.s. |
|  |  |  |  |  |  |  |  |  | Control vs. VAD: 18 | 0.9788 | n.s. |
| Figure 4J | Total visits after VA replenishment | Two-tailed unpaired t-test | 1 1 1 12 | F(1, 12) = 2.183 F(1, 12) = 0.000 F(1, 12) = 79.78 | 0.1653 >0.9999 <0.0001 | Diet | Control, VAD | Control: 4 VAD: 4 | Control vs. VAD: Success | 0.5332 | n.s. |
|  |  |  |  |  |  |  |  |  | Control vs. VAD: Failure | 0.5332 | n.s. |
| **Supplementary Figure 2** |  |  |  |  |  |  |  |  |  |  |  |
| Supplementary Figure 2A | DAPI+ cells in each blade of granule cells | Two-tailed unpaired t-test | 6 | F(3, 3) = 1.865 | 0.1684 | Blade of granule cell | Suprablade GC, infrablade GC | Suprablade GC: 4 Infrablade GC: 4 |  |  | n.s. |
| **Supplementary Figure 3** |  |  |  |  |  |  |  |  |  |  |  |
| Supplementary Figure 3A | Body weight measurement with dietary control | Mixed-effect model and post hoc Sidak's multiple comparisons tests | 1 | Time: F(11, 272) = 192.3 Diet: F(1, 32) = 0.03470 Time x Diet: F(11, 272) = 1.129 | <0.0001 0.8534 0.3381 | Dietary condition | Control, VAD | Control diet: 17 VAD diet: 17 | Control vs. VAD | >0.9 with all comparison groups | n.s. |
| Supplementary Figure 3B | RARβ mRNA level | Two-tailed unpaired t-test | 9 | F(3, 6) = 1.712 | <0.0001 | Dietary condition | Control, VAD | Control diet: 4 VAD diet: 7 | Control vs. VAD | <0.0001 | **** |
| **Supplementary Figure 4** |  |  |  |  |  |  |  |  |  |  |  |
| Supplementary Figure 4A | Food intake amount | Two-tailed unpaired t-test | 12 | F(6, 6) = 1.523 | 0.7573 | Dietary condition | Control, VAD | Control diet: 7 VAD diet: 7 | Control vs. VAD | 0.7573 | n.s. |
| Supplementary Figure 4B | Total exploration distance | Two-tailed unpaired t-test | 32 | F(16, 16) = 1.473 | 0.2009 | Dietary condition | Control, VAD | Control diet: 17 VAD diet: 17 | Control vs. VAD | 0.2009 | n.s. |
| Supplementary Figure 4C | Center zone exploration time in open field | Two-tailed unpaired t-test | 32 | F(16, 16) = 1.685 | 0.1588 | Dietary condition | Control, VAD | Control diet: 17 VAD diet: 17 | Control vs. VAD | 0.1588 | n.s. |
| Supplementary Figure 4D | Light zone exploration time in light & dark box | Two-tailed unpaired t-test | 32 | F(16, 16) = 1.428 | 0.5559 | Dietary condition | Control, VAD | Control diet: 17 VAD diet: 17 | Control vs. VAD | 0.5559 | n.s. |
| Supplementary Figure 4E | Open arm duration in elevated plus maze | Two-tailed unpaired t-test | 32 | F(16, 16) = 1.130 | 0.4089 | Dietary condition | Control, VAD | Control diet: 17 VAD diet: 17 | Control vs. VAD | 0.4089 | n.s. |
| Supplementary Figure 4G | Sucrose intake amount in sucrose preference test | Two-way ANOVA and post hoc Tukey’s multiple comparisons tests | 1 1 1 24 | Interaction: F(1, 24) = 3.063 Liquid: F(1, 24) = 77.06 Diet: F(1, 24) = 2.049 | 0.0928 <0.0001 0.1655 | Dietary condition, liquid | Control, VAD | Control diet: 7 VAD diet: 7 | Control water vs. Control 4% sucrose | 0.0002 | *** |
|  |  |  |  |  |  |  |  |  | Control water vs. VAD water | 0.9958 | n.s. |
|  |  |  |  |  |  |  |  |  | Control 4% sucrose vs. VAD 4% sucrose | 0.1388 | n.s. |
|  |  |  |  |  |  |  |  |  | VAD water vs. VAD 4% sucrose | <0.0001 | **** |
| Supplementary Figure 4H | Sucrose preference in sucrose preference test | Two-tailed unpaired t-test | 12 | F(6, 6) = 5.970 | 0.2095 | Dietary condition | Control, VAD | Control diet: 7 VAD diet: 7 |  | 0.2095 | n.s. |
